# Macrophage activation syndrome in juvenile dermatomyositis: a case report and a comprehensive review of the literature

**DOI:** 10.1186/s12969-023-00893-w

**Published:** 2023-09-21

**Authors:** Yong Chang, Xueyan Shan, Yongpeng Ge

**Affiliations:** 1 Department of Rheumatology, Tianshui Hospital of Traditional Chinese Medicine, Tianshui, China; 2 Department of Rheumatology, Guang’anmen Hospital, China Academy of Chinese Medical Sciences, Beijing, China; 3 Beijing University of Chinese Medicine, Beijing, China; 4 Department of Rheumatology, Key Laboratory of Myositis, China-Japan Friendship Hospital, Beijing, China

**Keywords:** Juvenile dermatomyositis, Macrophage activation syndrome, Hemophagocytic syndrome, Anti-nuclear matrix protein 2 (NXP2) antibody

## Abstract

**Background:**

Macrophage activation syndrome (MAS) is a severe and life-threatening syndrome associated with autoimmune diseases. The coexistence of MAS and juvenile dermatomyositis (JDM) is not well reported. This report describes a case of JDM with MAS and summarizes the clinical characteristics and prognosis of MAS in patients with JDM.

**Case presentation:**

The patient was a 15-year-old female with JDM, presenting with heliotrope rash, muscle weakness, increased muscle enzyme, anti-nuclear matrix protein 2 (NXP2) antibody, and muscle biopsy consistent with JDM. The patient developed fever, cytopenia, and hyperferritinemia three months after the first manifestations. Hemophagocytosis was found in the bone marrow. The final diagnosis was JDM combined with MAS. Despite intensive treatment, the patient died of MAS. By reviewing the literature, we found 17 similar cases. Together with the present case, 18 patients were identified, the median age of disease onset was 13.5 years, and male to female ratio was 1.25: 1. Nine out of 16 (56.3%) patients were complicated with interstitial lung disease (ILD). The median time interval between JDM onset and MAS diagnosis was 9 weeks. At the onset of MAS, all (100%) patients had elevated levels of ferritin and serum liver enzymes. Among 18 patients, 14 (77.8%) had fever, 14/17 (82.4%) had cytopenia, 11/11 (100%) had hepatosplenomegaly, and 13/14 (92.9%) had hemophagocytosis. Five (27.8%) patients showed central nervous system (CNS) involvement. The mortality of MAS rate of in patients with JDM was 16.7%, despite various treatment methods.

**Conclusion:**

. The coexistence of JDM and MAS is underestimated with increased mortality. Hepatosplenomegaly and increased serum levels of ferritin in patients with JDM should raise clinical suspicion for MAS.

**Supplementary Information:**

The online version contains supplementary material available at 10.1186/s12969-023-00893-w.

## Background

Juvenile dermatomyositis (JDM) is a subtype of idiopathic inflammatory myopathies (IIM), a group of diseases characterized by muscle inflammation and weakness. JDM presents with heliotrope rash and/or Gottron’s papules [1]. Hemophagocytic lymphohistiocytosis (HLH) or hemophagocytic syndrome (HPS) is a life-threatening syndrome associated with dysregulated hyperinflammatory response of lymphocytes and macrophages, resulting in fever, cytopenia, hepatosplenomegaly, and hyperferritinemia. It is a life-threatening complication of rheumatic diseases [2]. It is classically divided into primary or familial HLH/HPS and secondary HLH/HPS. HLH/HPS in the context of rheumatologic diseases is regarded as secondary HLH/HPS, commonly known as macrophage activation syndrome (MAS). It is a common complication of systemic juvenile idiopathic arthritis (sJIA) and adult-onset Still’s disease (AOSD) [3].

This study describes a patient with JDM who was complicated with MAS. The patient had anti-nuclear matrix protein 2 (NXP2) antibody, and despite treatment, the patient’s condition did not improve, and eventually died. Additionally, we searched the relevant literature and summarized the clinical characteristics of patients with JDM who were complicated with MAS.

## Case presentation

A 15-year-old female patient was admitted to our hospital due to skin lesions for more than 2 months, muscle weakness for 1 month, and dysphagia for 3 days before her visit.

Two months before admission, the patient experienced facial swelling and pain, red papules appeared on her eyelids. The local hospital treated her for allergy, but the symptoms did not improve. The weakness of proximal limbs and cervical flexor began one month before admission and was associated with upper limbs edema. Muscle weakness gradually progressed, and she could not do her daily activities such as tooth brushing, hair combing, and going up and down stairs. Laboratory examinations revealed elevated levels of serum creatine kinase (CK, 2914U/L; reference.

range: 26-200U/L) and lactate dehydrogenase (LDH, 555U/L; reference range: 100-250U/L). Erythrocyte sedimentation rate (ESR), C-reactive protein (CRP), and complete blood count were normal. Anti-nuclear matrix protein 2 (NXP2) antibody was found in her serum sample. Her pulmonary CT showed pleural thickening, a small amount of effusion, and subcutaneous edema of the chest wall. Echocardiography showed a small amount of pericardial effusion. Muscle biopsy revealed inflammatory cell infiltration, muscle fiber degeneration, necrosis and phagocytosis, and perivascular inflammatory infiltration. MHC-I staining was positive. She was diagnosed with JDM and subsequently given intravenous methylprednisolone (IVMP) 80 mg daily and intravenous immunoglobulin (IVIG) 20 g for 4 consecutive days in local hospital. Then, her skin rash was alleviated, and her muscle strength slightly improved. But dysphagia, choking cough during drinking, and hoarseness appeared 3 days before admission to our hospital.

On admission to our hospital, physical examinations revealed a body temperature of 37.1℃. Her only skin lesion was heliotrope rash. Mild edema of the upper arms was found in physical examination. Decreased muscle force was detected mainly in the proximal segment of the upper (Medical Research Council (MRC) Scale for muscle strength grade 3) and lower (MRC grade 2) extremities. Laboratory examinations revealed hyperferritinemia (443.3ng/mL; reference.

Range: 11-306ng/ml), and hyperCKemias (2495U/L). The serum levels of LDH (503U/L), aspartate aminotransferase (AST) (165U/L; reference range: 0-42U/L), alanine aminotransferase (ALT) (73U/L; reference range: 0-40U/L) and γ-glutamyltranspeptidase (GGT) (70U/L; reference range: 0-52U/L) were slightly elevated. Ro-52 antibody was positive.

She was treated with IVMP 40 mg (1 mg/kg) daily, intravenous cyclophosphamide (IVCYC) 400 mg (10 mg/kg) every two weeks, and tocilizumab, a humanized monoclonal interleukin (IL)-6 receptor inhibitor, 480 mg (8 mg/kg). The skin rash and subcutaneous edema were relieved, and muscle strength improved.

Three weeks later, the patient developed a high fever (39.1℃) and headache after catching a cold. Laboratory examinations revealed leukopenia, anemia, and thrombocytopenia. Epstein-Barr virus (EBV), cytomegalovirus (CMV), varicella zoster virus (VZV), or herpes simplex virus (HSV) was negative. The serum level of ferritin was 4322ng/mL. The serum level of CK decreased to 1545U/L, but the serum levels of ALT, AST, and LDH increased after treatment. Abdominal ultrasound showed splenomegaly. Bone marrow aspiration revealed haemophagocytosis. The diagnosis of MAS was established according to the HLH-2004 criteria [4]. Despite intensive treatment with dexamethasone, cyclosporin (CsA), and IVIG, her condition further deteriorated, and she died due to refractory MAS and multiple organ failure 6 weeks later.

## Literature review

### Methods

#### Search strategy

**Fig. 1:**
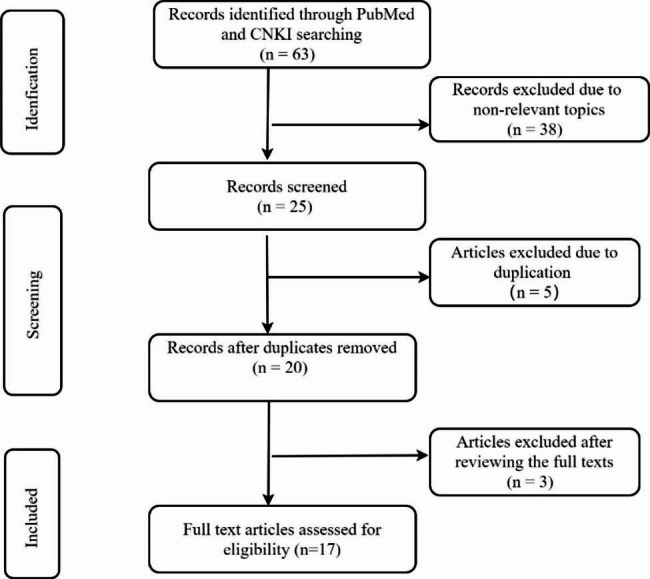
The article selection process in this systematic review

We systematically searched records from PubMed and China National Knowledge Infrastructure (CNKI) databases. Both English and Chinese literature was identified. Reference lists from each paper identified were hand searched. All articles from 2000 to December 2022 could be included. The following keywords were used for the search: juvenile dermatomyositis, macrophage activation syndrome, and hemophagocytic syndrome. We extracted data from patients with MAS or HPS or HLH. All articles were independently reviewed by two authors. The whole process is presented in Fig. 1.

## Results

Finally, 17 patients were identified through our literature search [5–21]. The characteristics of these patients are summarized in Tables 1 and 2. Together with the patient in our report, 18 patients were identified. Only 3 patients developed the disease below 10 years old, at 4, 5, and 7 years old. Other patients developed the disease after 10 years of age. The median age for disease onset was 13.5 years (range 4–17 years), and the male-to-female ratio was 1.25:1.

**Table 1:** The clinical features of JDM complicated with MAS

Patient number	Study (year)	Patient/ gender/age	Skin lesions	MS, and CK level (U/L)	Arthritis	ILD	MSA	From JDM onset to MAS
1	Kobayashi et al. (2000) [5]	1/M/14	Gottron’s papules and heliotrope eruption	Weakness; CK 26U/L	Yes	Yes	ND	3 months
2	Sterba et al. (2004) [6]	1/M/12	ND	ND	ND	ND	ND	ND
3	Kobayashi et al. (2006) [7]	1/F/10	Gottron’s papules and heliotrope eruption	Weakness; CK 579 U/L	No	Yes	ND	2 weeks
4	Yajima et al. (2008) [8]	1/M/16 ADM	ND	Normal	Yes	Yes	ND	Active phase of DM
5	Bustos et al. (2012) [9]	1/M/7	Heliotrope sign and facial edema	Weakness; CK 16,353 U/L	No	ND	ND	4 weeks
6	Wang et al. (2013) [10]	1/M/15,	Heliotrope sign and facial edema	Weakness; CK 3325 U/L	No	No	ND	2 weeks
7	Yamashita et al. (2013) [11]	1/F/17	Heliotrope, Gottron’s sign, and nail-fold lesions	Weakness; CK 1094 U/L	Yes	Yes	No	3 months
8	Lilleby et al. (2014) [12]	1/M/12	Facial erythema and facial swelling	Weakness; CK 14,000 U/L	No	Yes	ND	5 weeks
9	Ye et al. (2014) [13]	1/M/16	Heliotrope rash and facial swelling	Weakness; CK 1071 U/L	No	Yes	No	6 weeks
10	Poddighe et al. (2014) [14]	1/F/13 ADM	Heliotrope, Gottron’s rash, and peri-orbital edema	Normal	Yes	Yes	ND	3 months
11	Önen et al. (2014) [15]	1/F/12	Heliotrope rash	Weakness; CK 25 U/L	No	No	ND	6 months
12	Wakiguchi et al. (2015) [16]	1/F/4	Heliotrope rash and Gottron’s sign	Weakness; CK 40 U/L	No	Yes	MDA5	3 months
13	Teshigawara et al. (2016) [17]	1/M/17	Heliotrope eruption	Myalgia; CK 1869 U/L	No	No	No	6 months
14	Deschamps et al. (2018) [18]	1/F/17	Right facial swelling	Weakness; CK 4265 U/L	No	No	NXP2	5 weeks
15	Stewart JA et al. (2022) [19]	1/F/14	Ulcerative lesions on the dorsum of the hands	Weakness; CK 217 U/L	Yes	Yes	MDA5	3 months
16	Mouri M et al. (2022) [20]	1/M/5	Heliotrope rash and Gottron’s sign	Weakness; CK 2511 U/L	Yes	No	NXP2	2 weeks
17	Jagwani H et al. (2022) [21]	1/M/11	Heliotrope sign	Weakness; elevated CK level	ND	No	No	4 weeks
18	Our case	1/F/15	Heliotrope rash and mild edema on upper arm	Weakness, CK 2914 U/L	No	No	NXP2	3 months

ADM, amyopathic DM; F, female; M male; MSA, myositis-specific antibody; MS, muscle strength; ND, not described

**Table 2:** The clinical and laboratory characteristics of JDM patients at initial presentation of MAS

Patient number	Study (year)	Fever	Serum enzymes	Ferritin (ng/mL)	Blood cells	sIL-2R (U/ml)	HSM	HPC	Treatment and outcome
1	Kobayashi et al. (2000)	No	AST 309 U/L, LDH 752 U/L	765	WBC 3.4^*^10^9^/L, Hb 12.2 g/dL, PLT 7^*^10^9^/L	1120	Yes	Yes	Oral PSL, CsA, IVIG; improvement
2	Sterba et al. (2004)	Yes	ND	ND	Severe pancytopenia	ND	Yes	No	CS, CsA; improvement
3	Kobayashi et al. (2006)	Yes	AST 145 U/L, LDH 941 U/L	679	WBC 2.7^*^10^9^/L, Hb 14.6 g/dL, PLT 119^*^10^9^/L	1610	ND	Yes	IVMP, CsA; improvement
4	Yajima et al. (2008)	Yes	ND	2796	Neu 2.56^*^10^9^/L, Hb 7.7 g/dL, PLT 39^*^10^9^/L	882	Yes	Yes	IVMP, ISAs, IVIG; Death
5	Bustos et al. (2012).	Yes	ALT 241 U/L, AST 515 U/L, LDH 1537 U/L	1789	Cytopenia	ND	Yes	ND	IVMP, CsA, IVIG, PE; improvement
6	Wang et al. (2013)	Yes	ALT 64 U/L, AST 145 U/L, LDH 707 U/L	> 1500	WBC 2.02^*^10^9^/L, Hb 12.5 g/dL, PLT 84^*^10^9^/L	ND	Yes	Yes	IVMP/PSL, CYC; improvement
7	Yamashita et al. (2013)	Yes	AST 721 U/L, ALT 292 U/L, LDH 1069 U/L	4172	WBC 2.82^*^10^9^/L, Hb 8.6 g/dL, PLT 112^*^10^9^/L	2046	Yes	ND	IVDEX, CSA, CYC; Death (diffuse alveolar damage)
8	Lilleby et al. (2014)	Yes	ND	7437	ND	ND	ND	Yes	IVMP/PSL, IVIG, CSA, ETO, anakinra; improvement
9	Ye et al. (2014)	Yes	ALT 268 U/L, AST 147 U/L, LDH 964 U/L	8962	WBC 7.84^*^10^9^/L, Hb 14.2 g/dL, PLT 122^*^10^9^/L	ND	Yes	Yes	IVMP/DEX, CsA; improvement
10	Poddighe et al. (2014)	Yes	AST 50 U/L, ALT 14 U/L, LDH 890 U/L	5899	WBC 9.5^*^10^9^/L, Hb 11.7 g/dL, PLT 244^*^10^9^/L	ND	Yes	Yes	IVMP/oral PSL, CSA; improvement
11	Önen et al. (2014)	Yes	AST 205 U/L, ALT 59 U/L, LDH 1085 U/L	14,657	WBC 6.92^*^10^9^/L, Hb 8.4 g/dL, PLT 144^*^10^9^/L	ND	Yes	Yes	IVMP, CSA, IVIG; improvement
12	Wakiguchi et al. (2015)	Yes	AST1154 U/L, ALT 596 U/L, LDH 2267 U/L	8062	WBC 2.56^*^10^9^/L, Hb 13.4 g/dL, PLT 119^*^10^9^/L	ND	ND	Yes	IVMP/DEX, CsA, CYC; improvement
13	Teshigawara et al. (2016)	No	ND	967.4	WBC 3.9^*^10^9^/L, Hb 13.4 g/dL, PLT 124^*^10^9^/L	ND	ND	Yes	IVMP/DEX/, CYC, CsA/TAC; improvement
14	Deschamps et al. (2018)	Yes	ALT 245 U/L, AST 302 U/L, LDH 578 U/L	577	WBC 3.9^*^10^9^/L, Hb 10.7 g/dL, PLT 124^*^10^9^/L	ND	ND	Yes	IVMP/oral PSL, IVIG, CsA; improvement
15	Stewart JA et al. (2022)	Yes	ALT 157 U/L, AST 281 U/L, LDH 696 U/L	1641	Pancytopenia	ND	ND	ND	IVMP/DEX, Anakinra, IVIG, ETO, TOF; improvement
16	Mouri M et al. (2022)	No	ALT 795 U/L, AST 1608 U/L, LDH 925 U/L	619	WBC 2.8*10^9^/L, Hb 13.3 g/dL, PLT 69*10^9^/L	ND	Yes	Yes	IVMP, TAC, IVIG, CsA, CYC, PE; improvement
17	Jagwani H et al. (2022)	No	ND	4059	Pancytopenia	ND	ND	ND	IVMP, IVIG, CsA; improvement
18	Our case	Yes	ALT 153 U/L, AST 199 U/L, LDH 780 U/L	4322	WBC 2.8^*^10^9^/L, Hb 10.2 g/dL, PLT 85^*^10^9^/L	ND	Yes	Yes	IVMP, CsA; Death

CS, corticosteroids; CsA, cyclosporin; CYC, cyclophosphamide; DEX, dexamethasone; ETO, Etoposide; Hb, hemoglobin; HPC, Hemophagocytosis; HSM, hepatosplenomegaly; IVIG, intravenous immunoglobulin; IVMP, intravenous methylprednisolone; ND, not described; Neu, Neutrophils; PE, Plasma exchange; PLT, platelet; PSL, prednisolone; TAC, tacrolimus; TOF, tofacitinib; WBC, white blood cell

The main symptoms of JDM included cutaneous lesions in16/16 (100%), muscular weakness in15/17 (88.2%) and articular manifestations in 5/14 (35.7%) patients. In addition to the typical rash, 6/14 (42.9%) cases of JDM developed facial edema. Two cases of amyopathic JDM (AJDM) had no muscle weakness or abnormal CK levels. Including our case, myositis-specific antibodies were reported for 5 patients. Two cases of JDM had anti-melanoma differentiation-associated gene 5 (anti-MDA5) antibody and 3 cases had anti-NXP2 antibody. In addition, interstitial lung disease (ILD) was detected in 9/16 (56.3%) of patients by high-resolution CT (HRCT).

The median interval between the onset of JDM and MAS was 9 weeks (range: 2 weeks to 6 months, available from 16 patients). At the onset of MAS, most of the patients (77.8%, n = 14) had fever, 14/17 (82.4%) had leukopenia, anemia, or thrombocytopenia. All patients (100%) had elevated serum levels of ferritin, ALT, AST, and LDH. Hepatosplenomegaly was reported in 11/11 (100%) patients. Hemophagocytosis or macrophage aggregation was confirmed by bone marrow or lymph node biopsy in 13/14 (92.9%) cases of JDM. Five (27.8%) out of 18 patients showed central nervous system (CNS) involvement.

All patients received glucocorticoid and immunosuppressants (ISAs), such as CsA, CYC, and tacrolimus (TAC), and other treatments, such as IVIG, etoposide, plasma exchange (PE), and anakinra. Fifteen (83.3%) patients recovered; however, three (16.7%) patients died of MAS.

## Discussion

In this report, we presented a case of JDM with anti-NXP2 antibody and concomitant MAS which was confirmed by clinical symptoms and laboratory findings. We also reviewed the literature to include similar cases. In total, 18 cases with concomitant JDM and MAS have been reported to date. Regarding the temporal relationship, most of the patients developed MAS within three months after JDM diagnosis. Thus, MAS should not be neglected when JDM patients present with a high fever, cytopenia, and hyperferritinemia.

Our case was a typical case of JDM, characterized by skin rash, muscle weakness, and elevated levels of CK and NXP2 antibody. Muscle biopsy was consistent with JDM. Myositis-specific antibodies have emerged as valuable laboratory indicators for patients with equivocal clinical characteristics of dermatomyositis. NXP2 antibodies in JDM are often associated with calcinosis cutis and severe myopathy [22–24]. Patients with this type of JDM are more likely to have subcutaneous edema, which may be related to vasculopathy. During the initial treatment, our patient gradually developed dysphagia and choking cough. These findings show that her muscle weakness was severe and refractory to conventional treatment. Deschamps et al. reported a similar case, characterized by facial edema, proximal muscle weakness, dysphagia, hoarse voice, and MAS-related symptoms, which manifested 5 weeks after the diagnosis of JDM. Their patient was positive for NXP2 antibody [18].

The prevalence of MAS is from 0.9 to 4.6%, 7–13%, and 1–1.5% in patients with SLE, sJIA, and Kawasaki disease, respectively [25–27]. The systematic review published by Dimitri Poddighe et al. [28] indicated that the incidence rate of JDM is lower than that of SLE and JIA among children. However, the coincidence of JDM and MAS is only found in case reports. Therefore, the incidence of MAS in patients with JDM may have been underestimated.

Most frequently, secondary HLH or MAS was triggered by infections. In particular, infectious of especially EBV and other members of the Herpesvirus family, bacteria, and fungi are the triggers of this clinical picture. The patient had a history of upper respiratory tract infections before MAS, suggesting that MAS may be induced by infection. Although pay more attention to the infection, we did not find any evidence of pathogenic microbial infection, but this cannot rule out the involvement of infection factors.

In review of previous studies reporting the co-occurrence of JDM and MAS in the past 20 years, we found 18 patients, male-to-female ratio was 1.25:1. Most of the patients developed MAS after 10 years of age. Therefore, among patients with JDM who are older than 10 years, particular attention should be paid to fever and elevated serum levels of ferritin to avoid delay in the diagnosis and treatment of MAS.

The first manifestation of our patient was subcutaneous limb edema. Among other cases, 6 cases had facial or periorbital swelling or edema at the onset of the disease. Lilleby et al. suggested that facial swelling implies more severe JDM and MAS is more likely to develop in this type of JDM [12].

It has been reported that 14% of JDM patients have ILD [29], while nearly two-thirds of JDM patients with concomitant MAS have ILD, suggesting that JDM with ILD may be more prone to macrophage activation. Gupta et al. [30] reported that the incidence of central nervous system lesions in HLH/MAS associated with rheumatic disease was lower than that in HLH/MAS secondary to a tumor or virus. However, a CNS lesion indicates a severe MAS and poor prognosis. Four patients with CNS involvement were found in the literature review, and one patient died despite active treatment.

In addition, we identified two JDM patients with anti-MDA5 antibody developed MAS. A recent study showed that 2% of adult DM patients with anti-MDA5 antibody developed MAS, and half of patients with MDA5-positive DM and concomitant MAS had a poor prognosis [31]. Patients with anti-MDA5 had significantly higher serum levels of soluble CD163 (sCD163) than DM patients without such antibody [32]. CD163 is an exclusive marker of cells with monocyte/macrophage lineage. It is usually expressed on macrophages, and elevated levels of serum sCD163 have been reported as a marker of macrophage activation in various diseases such as MAS [33, 34]. Besides, patients with anti-MDA5 often have lymphopenia, increased serum levels of liver enzymes and hyperferritinemia, which may indicate that JDM patients with anti-MDA5 antibody are prone to MAS [35, 36].

Including this case, there were 3 cases of JDM with anti-NXP2 antibody. Mouri M et al. reported serum neopterin levels were elevated in a JDM with anti-NXP2 complicated by MAS [20]. One study demonstrated that the elevation of serum neopterin levels in patients with HLH was often very significant, and argued that serum neopterin levels very specific and sensitive for the diagnosis of HLH [37]. A retrospective study reported that the serum neopterin was elevated in nearly 80% of untreated JDM, and the group with anti-NXP2 antibody had the highest serum neopterin [38]. These evidences suggested that the JDM patients with anti-NXP2 antibody may be prone to complicate with MAS.

For this patient, we did not use methotrexate because previous experience had shown that methotrexate had a poor effect on JDM with anti-NXP2 antibody in our cohort (unpublished data). To reduce the risk of infection, we applied low-dose CYC. Furthermore, this patient was treated with tocilizumab due to previous study found that IL-6 monoclonal antibody could improve muscle weakness [39].

IVMP pulse therapy alone is insufficient for MAS in most cases. Despite aggressive combination therapy, three cases of JDM died. Kishida et al. reviewed adult DM complicated with MAS/HPS. Seven of the 18 patients (38.9%) died due to MAS/HPS or its complications [40]. Two JDM patients with MAS treated with interleukin-1 receptor antagonist anakinra in the reviewed literature [10, 17]. Other study demonstrated that anakinra appeared to be effective in treating pediatric patients with non-malignancy-associated secondary HLH/MAS, especially when it is given early in the disease course and when administered to patients who have an underlying rheumatic disease [41]. But unfortunately, IL-1 blockers were not available in our hospital.

## Conclusions

MAS is a serious complication of rheumatic diseases in children. The disease progresses rapidly with a high mortality rate. The co-incidence of JDM and MAS is underestimated and can be easily missed. Early diagnosis and active treatment play a key role in the prognosis of JDM. For JDM patients with fever, elevated serum levels of ferritin, hepatosplenomegaly, and hemocytopenia, MAS should be strongly suspected.

### Electronic supplementary material

Below is the link to the electronic supplementary material.

Supplementary Material 1

## Data Availability

The datasets for this article are not publicly available due to concerns regarding participant/patient anonymity. Requests to access the datasets should be directed to the corresponding author.

## Abbreviations

ALT: Alanine aminotransferase
AOSD: Adult-onset Still’s disease
AST: Aspartate aminotransferase
CK: Creatine kinase
CNKI: China National Knowledge Infrastructure
CNS: Central nervous system
CRP: C-reactive protein
CS: Corticosteroids
CsA: Cyclosporin
CYC: Cyclophosphamide
DEX: Dexamethasone
ETO: Etoposide
F: Female
GGT: Glutamyltranspeptidase
Hb: Hemoglobin
HLH: Hemophagocytic lymphohistiocytosis
HPC: Hemophagocytosis
HPS: Hemophagocytic syndrome
HRCT: High-resolution CT
HSM: Hepatosplenomegaly
IIM: Idiopathic inflammatory myopathies
ILD: Interstitial lung disease
ISAs: Immunosuppressants
IVIG: Intravenous immunoglobulin
JDM: Juvenile dermatomyositis
LDH: Lactate dehydrogenase
M: Male
MAS: Macrophage activation syndrome
MDA5: Melanoma differentiation-associated gene 5
MP: Methylprednisolone
MRC: Medical Research Council
MS: Muscle strength
MSA: Myositis-specific antibody
ND: Not described
NXP2: Nuclear matrix protein 2
PE: Plasma exchange
PLT: Platelet
PSL: Prednisolone
sJIA: Systemic juvenile idiopathic arthritis
SLE: Systemic lupus erythematosus
TAC: Tacrolimus
TOF: Tofacitinib
WBC: White blood cell
