# Supplementary material for: Macrophage activation syndrome in juvenile dermatomyositis: a case report and a comprehensive review of the literature
Source: Pediatr Rheumatol Online J. 2023 Sep 21;21:106. doi: 10.1186/s12969-023-00893-w (PMC10515226; doi:10.1186/s12969-023-00893-w)
Supplement: Supplementary file 1 — Supplementary Material 1 [file 12969_2023_893_MOESM1_ESM.docx]

**Macrophage activation syndrome in Juvenile dermatomyositis:** **a case report and a comprehensive review of the literature**

Yong Chang^1^, Xueyan Shan^2, 3^, Yongpeng Ge^4*^

^1^ Department of Rheumatology, Tianshui Hospital of Traditional Chinese Medicine

^2^ Rheumatology Department, Guang’anmen Hospital, China Academy of Chinese Medical Sciences, Beijing, China

^3^ Beijing University of Chinese Medicine, Beijing, China

^4^ Department of Rheumatology, Key Laboratory of Myositis, China-Japan Friendship Hospital

* Corresponding author: Yongpeng Ge

Department of Rheumatology, Key Laboratory of Myositis, China-Japan Friendship Hospital, Yinghua East Road, Chaoyang District, 100029, Beijing, China

E-mail: [gyp2016@163.com](mailto:gyp2016@163.com)

**Abstract**

**Background** Macrophage activation syndrome (MAS) is a severe and life-threatening syndrome associated with autoimmune diseases. The coexistence of MAS and juvenile dermatomyositis (JDM) is not well reported. This report describes a case of JDM with MAS and summarizes the clinical characteristics and prognosis of MAS in patients with JDM.

**Case presentation** The patient was a 15-year-old female with JDM, presenting with heliotrope rash, muscle weakness, increased muscle enzyme, anti-nuclear matrix protein 2 (NXP2) antibody, and muscle biopsy consistent with JDM. The patient developed fever, cytopenia, and hyperferritinemia three months after the first manifestations. Hemophagocytosis was found in the bone marrow. The final diagnosis was JDM combined with MAS. Despite intensive treatment, the patient died of MAS. By reviewing the literature, we found 17 similar cases. Together with the present case, 18 patients were identified, the median age of disease onset was 13.5 years, and male to female ratio was 1.25: 1. Nine out of 16 (56.3%) patients were complicated with interstitial lung disease (ILD). The median time interval between JDM onset and MAS diagnosis was 9 weeks. At the onset of MAS, all (100%) patients had elevated levels of ferritin and serum liver enzymes. Among 18 patients, 14 (77.8%) had fever, 14/17 (82.4%) had cytopenia, 11/11 (100%) had hepatosplenomegaly, and 13/14 (92.9%) had hemophagocytosis. Five (27.8%) patients showed central nervous system (CNS) involvement. The mortality of MAS rate of in patients with JDM was 16.7%, despite various treatment methods.

**Conclusion**. The coexistence of JDM and MAS is underestimated with increased mortality.. Hepatosplenomegaly and increased serum levels of ferritin in patients with JDM should raise clinical suspicion for MAS.

**Keywords:** Juvenile dermatomyositis; Macrophage activation syndrome; Hemophagocytic syndrome; Anti-nuclear matrix protein 2 (NXP2) antibody

**Background**

Juvenile dermatomyositis (JDM) is a subtype of idiopathic inflammatory myopathies (IIM), a group of diseases characterized by muscle inflammation and weakness. JDM presents with heliotrope rash and/or Gottron’s papules [1]. Hemophagocytic lymphohistiocytosis (HLH) or hemophagocytic syndrome (HPS) is a life-threatening syndrome associated with dysregulated hyperinflammatory response of lymphocytes and macrophages, resulting in fever, cytopenia, hepatosplenomegaly, and hyperferritinemia. It is a life-threatening complication of rheumatic diseases [2]. It is classically divided into primary or familial HLH/HPS and secondary HLH/HPS. HLH/HPS in the context of rheumatologic diseases is regarded as secondary HLH/HPS, commonly known as macrophage activation syndrome (MAS). It is a common complication of systemic juvenile idiopathic arthritis (sJIA) and adult-onset Still's disease (AOSD) [3].

This study describes a patient with JDM who was complicated with MAS. The patient had anti-nuclear matrix protein 2 (NXP2) antibody, and despite treatment, the patient's condition did not improve, and eventually died. Additionally, we searched the relevant literature and summarized the clinical characteristics of patients with JDM who were complicated with MAS.

**Case presentation**

A 15-year-old female patient was admitted to our hospital due to skin lesions for more than 2 months, muscle weakness for 1 month, and dysphagia for 3 days before her visit.

Two months before admission, the patient experienced facial swelling and pain, red papules appeared on her eyelids. The local hospital treated her for allergy, but the symptoms did not improve. The weakness of proximal limbs and cervical flexor began one month before admission and was associated with upper limbs edema. Muscle weakness gradually progressed, and she could not do her daily activities such as tooth brushing, hair combing, and going up and down stairs. Laboratory examinations revealed elevated levels of serum creatine kinase (CK, 2914U/L; reference

range: 26-200U/L) and lactate dehydrogenase (LDH, 555U/L; reference range: 100-250U/L). Erythrocyte sedimentation rate (ESR), C-reactive protein (CRP), and complete blood count were normal. Anti-nuclear matrix protein 2 (NXP2) antibody was found in her serum sample. Her pulmonary CT showed pleural thickening, a small amount of effusion, and subcutaneous edema of the chest wall. Echocardiography showed a small amount of pericardial effusion. Muscle biopsy revealed inflammatory cell infiltration, muscle fiber degeneration, necrosis, phagocytosis in blood vessels, and perivascular inflammatory infiltration. MHC-I staining was positive. She was diagnosed with JDM and subsequently given intravenous methylprednisolone (IVMP) 80 mg daily and intravenous immunoglobulin (IVIG) 20 g for 4 consecutive days in local hospital. Then, her skin rash was alleviated, and her muscle strength slightly improved. But dysphagia, choking cough during drinking, and hoarseness appeared 3 days before admission to our hospital.

On admission to our hospital, physical examinations revealed a body temperature of 37.1℃. Her only skin lesion was heliotrope rash. Mild edema of the upper arms was found in physical examination. Decreased muscle force was detected mainly in the proximal segment of the upper (Medical Research Council (MRC) Scale for muscle strength grade 3) and lower (MRC grade 2) extremities. Laboratory examinations revealed hyperferritinemia (443.3 ng/mL; reference

Range: 11-306 ng/ml), and hyperCKemias (2495 U/L). The serum levels of LDH (503 U/L), aspartate aminotransferase (AST) (165 U/L; reference range: 0-42U/L), alanine aminotransferase (ALT) (73 U/L; reference range: 0-40U/L) and γ-glutamyltranspeptidase (GGT) (70 U/L; ; reference range: 0-52U/L) were slightly elevated. Ro-52 antibody was positive.

She was treated with IVMP 40 mg (1 mg/kg) daily, intravenous cyclophosphamide (IVCYC) 400 mg (10 mg/kg) every two weeks, and tocilizumab, a humanized monoclonal interleukin (IL)-6 receptor inhibitor, 480 mg (8 mg/kg). The skin rash and subcutaneous edema were relieved, and muscle strength improved.

Three weeks later, the patient developed a high fever (39.1℃) and headache after catching a cold. Laboratory examinations revealed leukopenia, anemia, and thrombocytopenia. Epstein-Barr virus (EBV), cytomegalovirus (CMV), varicella zoster virus (VZV), or herpes simplex virus (HSV) was negative. The serum level of ferritin was 4322 ng/mL. The serum level of CK decreased to 1545 U/L, but the serum levels of ALT, AST, and LDH increased after treatment. Abdominal ultrasound showed splenomegaly. Bone marrow aspiration revealed haemophagocytosis. The diagnosis of MAS was established according to the HLH-2004 criteria [4]. Despite intensive treatment with dexamethasone, cyclosporin (CsA), and IVIG, her condition further deteriorated, and she died due to refractory MAS and multiple organ failure 6 weeks later.

**Literature review**

**Methods**

Search strategy

We systematically searched records from PubMed, and China National Knowledge Infrastructure (CNKI) databases. Both English and Chinese literature was identified. Reference lists from each paper identified were hand searched. All articles from 2000 to December 2022 could be included. The following keywords were used for the search: juvenile dermatomyositis, macrophage activation syndrome, and hemophagocytic syndrome. We extracted data from patients with MAS or HPS or HLH. All articles were independently reviewed by two authors. The whole process is presented in **Fig. 1**.


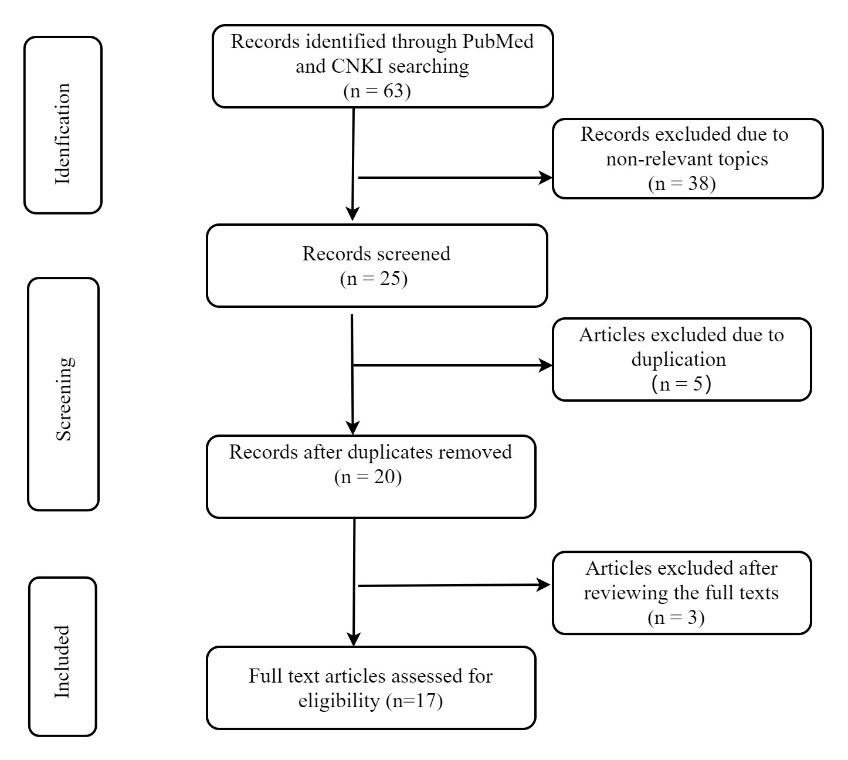


**Fig. 1** The article selection process in this systematic review.

**Results**

Finally, 17 patients were identified through our literature search [5-21]. The characteristics of these patients are summarized in **Table 1** and **Table 2**. Together with the patient in our report, 18 patients were identified. Only 3 patients developed the disease below 10 years old, at 4, 5, and 7 years old. Other patients developed the disease after 10 years of age. The median age for disease onset was 13.5 years (range 4-17 years), and the male-to-female ratio was 1.25:1.

The main symptoms of JDM included cutaneous lesions in16/16 (100%), muscular weakness in15/17 (88.2%) and articular manifestations in 5/14 (35.7%) patients. In addition to the typical rash, 6/14 (42.9%) cases of JDM developed facial edema. Two cases of amyopathic JDM (AJDM) had no muscle weakness or abnormal CK levels. Including our case, myositis-specific antibodies were reported for 5 patients. Two cases of JDM had anti-melanoma differentiation-associated gene 5 (anti-MDA5) antibody and 3 cases had anti-NXP2 antibody. In addition, interstitial lung disease (ILD) was detected in 9/16 (56.3%) of patients by high-resolution CT (HRCT).

The median interval between the onset of JDM and MAS was 9 weeks (range: 2 weeks to 6 months, available from 16 patients). At the onset of MAS, most of the patients (77.8%, n = 14) had fever, 14/17 (82.4%) had leukopenia, anemia, or thrombocytopenia. All patients (100%) had elevated serum levels of ferritin, ALT, AST, and LDH. Hepatosplenomegaly was reported in 11/11 (100%) patients. Hemophagocytosis or macrophage aggregation was confirmed by bone marrow or lymph node biopsy in 13/14 (92.9%) cases of JDM. Five (27.8%) out of 18 patients showed central nervous system (CNS) involvement.

All patients received glucocorticoid and immunosuppressants (ISAs), such as CsA, CYC, and tacrolimus (TAC), and other treatments, such as IVIG, etoposide, plasma exchange (PE), and anakinra. Fifteen (83.3%) patients recovered; however, three (16.7%) patients died of MAS.

**Discussion**

In this report, we presented a case of JDM with anti-NXP2 antibody and concomitant MAS which was confirmed by clinical symptoms and laboratory findings. We also reviewed the literature to include similar cases. In total, 18 cases with concomitant JDM and MAS have been reported to date. Regarding the temporal relationship, most of the patients developed MAS within three months after JDM diagnosis. Thus, MAS should not be neglected when JDM patients present with a high fever, cytopenia, and hyperferritinemia.

Our case was a typical case of JDM, characterized by skin rash, muscle weakness, and elevated levels of CK and NXP2 antibody. Muscle biopsy was consistent with JDM. Myositis-specific antibodies have emerged as valuable laboratory indicators for patients with equivocal clinical characteristics of dermatomyositis. NXP2 antibodies in JDM are often associated with calcinosis cutis and severe myopathy [22-24]. Patients with this type of JDM are more likely to have subcutaneous edema, which may be related to vasculopathy. During the initial treatment, our patient gradually developed dysphagia and choking cough. These findings show that her muscle weakness was severe and refractory to conventional treatment. Deschamps *et al*. reported a similar case, characterized by facial edema, proximal muscle weakness, dysphagia, hoarse voice, and MAS-related symptoms, which manifested 5 weeks after the diagnosis of JDM. Their patient was positive for NXP2 antibody [18].

The prevalence of MAS is from 0.9% to 4.6%, 7% to 13%, and 1% to 1.5% in patients with SLE, sJIA, and Kawasaki disease, respectively [25-27]. The systematic review published by Dimitri Poddighe *et al.* [28] indicated that the incidence rate of JDM is lower than that of SLE and JIA among children. However, the coincidence of JDM and MAS is only found in case reports. Therefore, the incidence of MAS in patients with JDM may have been underestimated.

Most frequently, secondary HLH or MAS was triggered by infections. In particular, infectious of especially EBV and other members of the Herpesvirus family, bacteria, and fungi are the triggers of this clinical picture. The patient had a history of upper respiratory tract infections before MAS, suggesting that MAS may be induced by infection. Although pay more attention to the infection, we did not find any evidence of pathogenic microbial infection, but this cannot rule out the involvement of infection factors.

In review of previous studies reporting the co-occurrence of JDM and MAS in the past 20 years, we found 18 patients, male-to-female ratio was 1.25:1. Most of the patients developed MAS after 10 years of age. Therefore, among patients with JDM who are older than 10 years, particular attention should be paid to fever and elevated serum levels of ferritin to avoid delay in the diagnosis and treatment of MAS.

The first manifestation of our patient was subcutaneous limb edema. Among other cases, 6 cases had facial or periorbital swelling or edema at the onset of the disease. Lilleby *et al.* suggested that facial swelling implies more severe JDM and MAS is more likely to develop in this type of JDM [12].

It has been reported that 14% of JDM patients have ILD [29], while nearly two-thirds of JDM patients with concomitant MAS have ILD, suggesting that JDM with ILD may be more prone to macrophage activation. Gupta *et al.* [30] reported that the incidence of central nervous system lesions in HLH/MAS associated with rheumatic disease was lower than that in HLH/MAS secondary to a tumor or virus. However, a CNS lesion indicates a severe MAS and poor prognosis. Four patients with CNS involvement were found in the literature review, and one patient died despite active treatment.

In addition, we identified two JDM patients with anti-MDA5 antibody developed MAS. A recent study showed that 2% of adult DM patients with anti-MDA5 antibody developed MAS, and half of patients with MDA5-positive DM and concomitant MAS had a poor prognosis [31].

Patients with anti-MDA5 had significantly higher serum levels of soluble CD163 (sCD163) than DM patients without sCD163 [32]. CD163 is an exclusive marker of cells with monocyte/macrophage lineage. It is usually expressed on macrophages, and elevated levels of serum sCD163 have been reported as a marker of macrophage activation in various diseases such as MAS [33-34]. Besides, patients with anti-MDA5 often have lymphopenia, increased serum levels of liver enzymes and hyperferritinemia, which may indicate that JDM patients with anti-MDA5 antibody are prone to MAS [35-36].

Including this case, there were 3 cases of JDM with antiNXP2 antibody. Mouri M *et al.* reported serum neopterin levels were elevated in a JDM with anti-NXP2 complicated by MAS [20]. One study demonstrated that the elevation of serum neopterin levels in patients with HLH was often very significant, and argued that serum neopterin levels very specific and sensitive for the diagnosis of HLH [37]. A retrospective study that the serum neopterin was elevated in nearly 80% of untreated JDM, and the group with anti-NXP2 antibody had the highest serum neopterin [38]. These evidences suggested that the JDM patients with anti-NXP2 antibody may be prone to complicate with MAS.

For this patient, we did not use methotrexate because previous experience had shown that methotrexate has a poor effect on JDM with anti-NXP2 antibody in our cohort (unpublished data). To reduce the risk of infection, we applied low-dose CYC. Furthermore, this patient was treated with tocilizumab due to previous study found that IL-6 monoclonal antibody could improve muscle weakness [39].

IVMP pulse therapy alone is insufficient for MAS in most cases. Despite aggressive combination therapy, three cases of JDM died. Kishida *et al.* reviewed adult DM complicated with MAS/HPS. Seven of the 18 patients (38.9%) died due to MAS/HPS or its complications [40]. Two JDM patients with MAS treated with interleukin-1 receptor antagonist anakinra in the reviewed literature [10, 17]. Other study demonstrated that anakinra appeared to be effective in treating pediatric patients with non-malignancy-associated secondary HLH/MAS, especially when it is given early in the disease course and when administered to patients who have an underlying rheumatic disease [41]. But unfortunately, IL-1 blockers were not available in our hospital.

**Conclusions**

MAS is a serious complication of rheumatic diseases in children. The disease progresses rapidly with a high mortality rate. The co-incidence of JDM and MAS is underestimated and can be easily missed. Early diagnosis and active treatment play a key role in the prognosis of JDM. For JDM patients with fever, elevated serum levels of ferritin, hepatosplenomegaly, and hemocytopenia, MAS should be strongly suspected.

**Abbreviations**

| ALT | Alanine aminotransferase |
| --- | --- |
| AOSD | Adult-onset Still's disease |
| AST | Aspartate aminotransferase |
| CK | Creatine kinase |
| CNKI | China National Knowledge Infrastructure |
| CNS | Central nervous system |
| CRP | C-reactive protein |
| CS | Corticosteroids |
| CsA | Cyclosporin |
| CYC | Cyclophosphamide |
| DEX | Dexamethasone |
| ETO | Etoposide |
| F | Female |
| GGT | Glutamyltranspeptidase |
| Hb | Hemoglobin |
| HLH | Hemophagocytic lymphohistiocytosis |
| HPC | Hemophagocytosis |
| HPS | Hemophagocytic syndrome |
| HRCT | High-resolution CT |
| HSM | Hepatosplenomegaly |
| IIM | Idiopathic inflammatory myopathies |
| ILD | Interstitial lung disease |
| ISAs | Immunosuppressants |
| IVIG | Intravenous immunoglobulin |
| JDM | Juvenile dermatomyositis |
| LDH | Lactate dehydrogenase |
| M | Male |
| MAS | Macrophage activation syndrome |
| MDA5 | Melanoma differentiation-associated gene 5 |
| MP | Methylprednisolone |
| MRC | Medical Research Council |
| MS | Muscle strength |
| MSA | Myositis-specific antibody |
| ND | Not described |
| NXP2 | Nuclear matrix protein 2 |
| PE | Plasma exchange |
| PLT | Platelet |
| PSL | Prednisolone |
| sJIA | Systemic juvenile idiopathic arthritis |
| SLE | Systemic lupus erythematosus |
| TAC | Tacrolimus |
| TOF | Tofacitinib |
| WBC | White blood cell |

**Table 1** The clinical features of JDM complicated with MAS

| Patient number | Study (year) | Patient/  gender/age | Skin lesions | MS, and CK level (U/L) | Arthritis | ILD | MSA | From JDM  onset to MAS |
| --- | --- | --- | --- | --- | --- | --- | --- | --- |
| 1 | Kobayashi *et al*. (2000) [5] | 1/M/14 | Gottron’s papules and heliotrope eruption | Weakness;  CK 26U/L | Yes | Yes | ND | 3 months |
| 2 | Sterba *et al*. (2004) [6] | 1/M/12 | ND | ND | ND | ND | ND | ND |
| 3 | Kobayashi *et al*. (2006) [7] | 1/F/10 | Gottron’s papules and heliotrope eruption | Weakness;  CK 579 U/L | No | Yes | ND | 2 weeks |
| 4 | Yajima *et al*.  (2008) [8] | 1/M/16 ADM | ND | Normal | Yes | Yes | ND | Active phase of DM |
| 5 | Bustos *et al*.  (2012) [9] | 1/M/7 | Heliotrope sign and facial edema | Weakness;  CK 16353 U/L | No | ND | ND | 4 weeks |
| 6 | Wang *et al*. (2013) [10] | 1/M/15, | Heliotrope sign and facial edema | Weakness;  CK 3325 U/L | No | No | ND | 2 weeks |
| 7 | Yamashita  *et al.* (2013) [11] | 1/F/17 | Heliotrope, Gottron’s sign, and nail-fold lesions | Weakness;  CK 1094 U/L | Yes | Yes | No | 3 months |
| 8 | Lilleby *et al* (2014) [12] | 1/M/12 | Facial erythema and facial swelling | Weakness;  CK 14000 U/L | No | Yes | ND | 5 weeks |
| 9 | Ye *et al.* (2014) [13] | 1/M/16 | Heliotrope rash and facial swelling | Weakness;  CK 1071 U/L | No | Yes | No | 6 weeks |
| 10 | Poddighe  *et al.* (2014) [14] | 1/F/13  ADM | Heliotrope, Gottron's rash, and peri-orbital edema | Normal | Yes | Yes | ND | 3 months |
| 11 | Önen *et al.*  (2014) [15] | 1/F/12 | Heliotrope rash | Weakness;  CK 25 U/L | No | No | ND | 6 months |
| 12 | Wakiguchi  *et al.* (2015) [16] | 1/F/4 | Heliotrope rash and Gottron's sign | Weakness;  CK 40 U/L | No | Yes | MDA5 | 3 months |
| 13 | Teshigawara *et al.* (2016) [17] | 1/M/17 | Heliotrope eruption | Myalgia;  CK 1869 U/L | No | No | No | 6 months |
| 14 | Deschamps *et al.* (2018) [18] | 1/F/17 | Right facial swelling | Weakness;  CK 4265 U/L | No | No | NXP2 | 5 weeks |
| 15 | Stewart JA *et al.* (2022) [19] | 1/F/14 | Ulcerative lesions on the dorsum of the hands | Weakness;  CK 217 U/L | Yes | Yes | MDA5 | 3 months |
| 16 | Mouri M *et al.* (2022) [20] | 1/M/5 | Heliotrope rash and Gottron's sign | Weakness;  CK 2511 U/L | Yes | No | NXP2 | 2 weeks |
| 17 | Jagwani H *et al.* (2022) [21] | 1/M/11 | Heliotrope sign | Weakness;  elevated CK level | ND | No | No | 4 weeks |
| 18 | Our case | 1/F/15 | Heliotrope rash and mild edema on upper arm | Weakness,  CK 2914 U/L | No | No | NXP2 | 3 months |

F, female; M male; MSA, myositis-specific antibody; MS, muscle strength; ND, not described.

**Table 2** The clinical and laboratory characteristics of JDM patients at initial presentation of MAS

| Patient number | Study (year) | Fever | Serum enzymes | Ferritin (ng/mL) | Blood cells | sIL-2R | HSM | HPC | Treatment and outcome |
| --- | --- | --- | --- | --- | --- | --- | --- | --- | --- |
| 1 | Kobayashi *et al*. (2000) | No | AST 309 U/L,  LDH 752 U/L | 765 | WBC 3.4^*^10^9^/L, Hb 12.2 g/dL, PLT 7^*^10^9^/L | 1120U/ml | Yes | Yes | Oral PSL, CsA, IVIG;  improvement |
| 2 | Sterba *et al*. (2004) | Yes | ND | ND | Severe pancytopenia | ND | Yes | No | CS, CsA;  improvement |
| 3 | Kobayashi *et al*. (2006) | Yes | AST 145 U/L,  LDH 941 U/L | 679 | WBC 2.7^*^10^9^/L, Hb 14.6 g/dL, PLT 119^*^10^9^/L | 1610U/ml | ND | Yes | IVMP, CsA;  improvement |
| 4 | Yajima *et al*.  (2008) | Yes | ND | 2796 | Neu 2.56^*^10^9^/L, Hb 7.7 g/dL, PLT 39^*^10^9^/L | 882U/ml | Yes | Yes | IVMP, ISAs, IVIG；  Death |
| 5 | Bustos *et al*.  (2012)*.* | Yes | ALT 241 U/L, AST 515 U/L, LDH 1537 U/L | 1789 | Cytopenia | ND | Yes | ND | IVMP, CsA, IVIG, PE; improvement |
| 6 | Wang *et al*. (2013) | Yes | ALT 64 U/L, AST 145 U/L, LDH 707 U/L | > 1500 | WBC 2.02^*^10^9^/L, Hb 12.5 g/dL, PLT 84^*^10^9^/L | ND | Yes | Yes | IVMP/PSL, CYC;  improvement |
| 7 | Yamashita  *et al*. (2013) | Yes | AST 721 U/L, ALT 292 U/L, LDH 1069 U/L | 4172 | WBC 2.82^*^10^9^/L, Hb 8.6 g/dL, PLT 112^*^10^9^/L | 2046U/ml | Yes | ND | IVDEX, CSA, CYC; Death (diffuse alveolar damage) |
| 8 | Lilleby *et al*. (2014) | Yes | ND | 7437 | ND | ND | ND | Yes | IVMP/PSL, IVIG, CSA, ETO, anakinra; improvement |
| 9 | Ye *et al*. (2014) | Yes | ALT 268 U/L, AST  147 U/L, LDH 964 U/L | 8962 | WBC 7.84^*^10^9^/L, Hb 14.2 g/dL, PLT 122^*^10^9^/L | ND | Yes | Yes | IVMP/DEX, CsA;  improvement |
| 10 | Poddighe  *et al*. (2014) | Yes | AST 50 U/L, ALT 14 U/L, LDH 890 U/L | 5899 | WBC 9.5^*^10^9^/L, Hb 11.7 g/dL, PLT 244^*^10^9^/L | ND | Yes | Yes | IVMP/oral PSL, CSA;  improvement |
| 11 | Önen *et al*.  (2014) | Yes | AST 205 U/L, ALT 59 U/L, LDH 1085 U/L | 14657 | WBC 6.92^*^10^9^/L, Hb 8.4 g/dL, PLT 144^*^10^9^/L | ND | Yes | Yes | IVMP, CSA, IVIG;  improvement |
| 12 | Wakiguchi  *et al*. (2015) | Yes | AST1154 U/L, ALT 596 U/L, LDH 2267 U/L | 8062 | WBC 2.56^*^10^9^/L, Hb 13.4 g/dL, PLT 119^*^10^9^/L | ND | ND | Yes | IVMP/DEX, CsA, CYC;  improvement |
| 13 | Teshigawara *et al*. (2016) | No | ND | 967.4 | WBC 3.9^*^10^9^/L, Hb 13.4 g/dL, PLT 124^*^10^9^/L | ND | ND | Yes | IVMP/DEX/, CYC，CsA/TAC;  improvement |
| 14 | Deschamps *et al*. (2018) | Yes | ALT 245 U/L, AST  302 U/L, LDH 578 U/L | 577 | WBC 3.9^*^10^9^/L, Hb 10.7 g/dL, PLT 124^*^10^9^/L | ND | ND | Yes | IVMP/oral PSL, IVIG, CsA; improvement |
| 15 | Stewart JA *et al*. (2022) | Yes | ALT 157 U/L, AST  281 U/L, LDH 696 U/L | 1641 | Pancytopenia | ND | ND | ND | IVMP/DEX, Anakinra, IVIG, ETO, TOF; improvement |
| 16 | Mouri M *et al.* (2022) | No | ALT 795 U/L, AST  1608 U/L, LDH 925 U/L | 619 | WBC 2.8*10^9^/L, Hb 13.3 g/dL, PLT 69*10^9^/L | ND | Yes | Yes | IVMP, TAC, IVIG, CsA, CYC, PE;  improvement |
| 17 | Jagwani H *et al.* (2022) | No | ND | 4059 | Pancytopenia | ND | ND | ND | IVMP, IVIG, CsA；  improvement |
| 18 | Our case | Yes | ALT 153 U/L, AST 199 U/L, LDH 780 U/L | 4322 | WBC 2.8^*^10^9^/L, Hb 10.2 g/dL, PLT 85^*^10^9^/L | ND | Yes | Yes | IVMP, CsA;  Death () |

CS, corticosteroids; CsA, cyclosporin; CYC, cyclophosphamide; DEX, dexamethasone; ETO, Etoposide; Hb, hemoglobin; HPC, Hemophagocytosis; HSM, hepatosplenomegaly; IVIG, intravenous immunoglobulin; IVMP, intravenous methylprednisolone; ND, not described; Neu, Neutrophils; PE, Plasma exchange; PLT, platelet; PSL, prednisolone; TAC, tacrolimus; TOF, tofacitinib; WBC, white blood cell.

**Acknowledgements**

The authors would like to express their gratitude to EditSprings (https://www.editsprings.com/) for the expert linguistic services provided.

**Authors’ contributions**

YC and YG wrote the main text of the case report. YC, XS and YG contributed to the conception and design of the case report. All authors have read and approved the manuscript.

**Availability of data and materials**

The datasets for this article are not publicly available due to concerns regarding participant/patient anonymity. Requests to access the datasets should be directed to the corresponding author.

**Funding**

This study was supported by the National High Level Hospital Clinical Research Funding (2022-NHLHCRF-YS-02) and Elite Medical Professionals Project of China-Japan Friendship Hospital (NO. ZRJY2023-GG02)

**Declarations**

**Ethics approval and consent to participate**

The Research Ethical Committee of China-Japan Friendship Hospital accredited this case study. We also obtained informed consent from the parents of the patient.

**Consent for publication**

We obtained written informed consent from the patient’s legal guardian to publish this case report.

**Competing interests**

The authors declare that there is no conflict of interest.

**References**

1. Kobayashi I, Akioka S, Kobayashi N, Iwata N, Takezaki S, Nakaseko H, et al. Clinical practice guidance for juvenile dermatomyositis (JDM) 2018-Update. Mod Rheumatol. 2020;30(3):411-423.

2. Esteban YM, de Jong JLO, Tesher MS. An Overview of Hemophagocytic Lymphohistiocytosis. Pediatr Ann. 2017;46(8): e309-e313.

3. Sen ES, Clarke SL, Ramanan AV. Macrophage Activation Syndrome. Indian J Pediatr. 2016; 83(3): 248-53.

4. Henter JI, Horne A, Aricó M, Egeler RM, Filipovich AH, Imashuku S, et al. HLH-2004: diagnostic and therapeutic guidelines for hemophagocytic lymphohistiocytosis. Pediatr Blood Cancer. 2007;48(2):124-131.

5. Kobayashi I, Yamada M, Kawamura N, Kobayashi R, Okano M, Kobayashi K. Platelet-specific hemophagocytosis in a patient with juvenile dermatomyositis. Acta Paediatr.2000;89(5):617-9.

6. Sterba G, Rodriguez C, Sifontes S, Vigilanza P. Macrophage activation syndrome due to methotrexate in a 12-year-old boy with dermatomyositis. J Rheumatol. 2004;31(5):1014–5.

7. Kobayashi I, Kawamura N, Okano M, Sageshima S, Nakayama T, Ohyu J, et al. Thrombocytopaenia in juvenile dermatomyositis. Scand J Rheumatol. 2006;35(1):79–80.

8. Yajima N, Wakabayashi K, Odai T, Isozaki T, Matsunawa M, Miwa Y, et al. Clinical features of hemophagocytic syndrome in patients with dermatomyositis. J Rheumatol. 2008;35(9):1838–41.

9. Bustos B R, Carrasco A C, Toledo R C. Plasmapheresis for macrophage activation syndrome and multiorgan failure as first presentation of juvenile dermatomyositis. An Pediatr (Barc). 2012;77(1):47–50.

10. Qianhan W, Qihua F, Jiang C, et al. A case of juvenile dermatomyositis complicated with macrophage activation syndrome. Chin J Clinicians (Electronic Edition). 2013, 7 (9): 4142-4143.

11. Yamashita H, Matsuki Y, Shimizu A, Mochizuki M, Takahashi Y, Kano T, et al. Hemophagocytic lymphohistiocytosis complicated by central nervous system lesions in a patient with dermatomyositis: a case presentation and literature review. Mod Rheumatol. 2013;23(2):386–92.

12. Lilleby V, Haydon J, Sanner H, Krossness BK, Ringstad G, Flatø B. Severe macrophage activation syndrome and central nervous system involvement in juvenile dermatomyositis. Scand J Rheumatol. 2014;43(2):171–3.

13. Bin Y, Xiaoxuan S, Chunxia Z, et al. Dermatomyositis complicated with hemophagocytic syndrome with normal peripheral blood system: a case report. Chin J Rheumatol. 2014, 18(9): 649-650.

14. Poddighe D, Cavagna L, Brazzelli V, Bruni P, Marseglia GL. A hyper-ferritinemia syndrome evolving in recurrent macrophage activation syndrome, as an onset of amyopathic juvenile dermatomyositis: a challenging clinical case in light of the current diagnostic criteria. Autoimmun Rev. 2014;13(11):1142–8.

15. Önen ŞŞ, Ümit Z, Siviş ZÖ, Evin F, Kasap M, Yaşar BE, et al. Juvenile Dermatomyositis with a Rare and Severe Complication: Macrophage Activation Syndrome. J Pediatr Res. 2014;1(4):218–21.

16. Wakiguchi H, Hasegawa S, Hirano R, Kaneyasu H, WakabayashiTakahara M, Ohga S. Successful control of juvenile dermatomyositis-associated macrophage activation syndrome and interstitial pneumonia: distinct kinetics of interleukin-6 and -18 levels. Pediatr Rheumatol Online J. 2015;13: 49.

17. Teshigawara S, Katada Y, Maeda Y, Yoshimura M, Kudo-Tanaka E, Tsuji S, et al. Hemophagocytic lymphohistiocytosis with leukoencephalopathy in a patient with dermatomyositis accompanied with peripheral T-cell lymphoma: a case report. J Med Case Rep. 2016, 2;10: 212.

18. Deschamps R, Couture J, Hadjinicolaou A, LeBlanc CMA. A 17-year-old girl with facial edema and weakness. Paediatr Child Health. 2018;23(4):241–4.

19. Stewart JA, Price T, Moser S, Mullikin D, Bryan A. Progressive, refractory macrophage activation syndrome as the initial presentation of anti-MDA5 antibody positive juvenile dermatomyositis: a case report and literature review. Pediatr Rheumatol Online J. 2022;20(1):16.

20. Mouri M, Kanamori T, Tanaka E, Hiratoko K, Okubo M, Inoue M, et al. Hepatic veno-occlusive disease accompanied by thrombotic microangiopathy developing during treatment of juvenile dermatomyositis and macrophage activation syndrome: A case report. Mod Rheumatol Case Rep. 2022; rxac086.

21. Jagwani H, Mondal A, Pal P. Juvenile Dermatomyositis With Macrophage Activation and Severe Encephalopathy. Indian Pediatr. 2022;59(9):725-6.

22. Tansley SL, Betteridge ZE, Shaddick G, Gunawardena H, Arnold K, Wedderburn LR, et al. Calcinosis in juvenile dermatomyositis is influenced by both anti-NXP2 autoantibody status and age at disease onset. Rheumatology (Oxford). 2014;53: 2204-8.

23. Wu Q, Wedderburn LR, McCann LJ. Juvenile dermatomyositis: Latest advances. Best Pract Res Clin Rheumatol. 2017; 31(4):535–57.

24. Gunawardena H, Wedderburn LR, Chinoy H, Betteridge ZE, North J, Ollier WER, et al. Autoantibodies to a 140-kd protein in juvenile dermatomyositis are associated with calcinosis. Arthritis Rheum. 2009;60(6):1807–14.

25. Vilaiyuk S, Sirachainan N, Wanitkun S, Pirojsakul K, Vaewpanich J. Recurrent macrophage activation syndrome as the primary manifestation in systemic lupus erythematosus and the benefit of serial ferritin measurements: a case-based review. Clin Rheumatol. 2013;32(6): 899-904.

26. Boom V, Anton J, Lahdenne P, Quartier P, Ravelli A, Wulffraat NM, et al. Evidenced-based diagnosis and treatment of macrophage activation syndrome in systemic juvenile idiopathic arthritis. Pediatr Rheumatol Online J. 2015;13: 55.

27. Wang W, Gong F, Zhu W, Fu S, Zhang Q. Macrophage activation syndrome in Kawasaki disease: more common than we thought? Semin Arthritis Rheum 2015;44(4):405-10.

28. Poddighe D, Dauyey K. Macrophage activation syndrome in juvenile dermatomyositis: a systematic review. Rheumatol Int. 2020;40(5):695-702.

29. Pouessel G, Deschildre A, Le Bourgeois M, Cuisset JM, Catteau B, Karila C, et al. The lung is involved in juvenile dermatomyositis. Pediatr Pulmonol. 2013;48(10):1016-25.

30. Gupta AA, Tyrrell P, Valani R, Benseler S, Abdelhaleem M, Weitzman S. Experience with hemophagocytic lymphohistiocytosis/macrophage activation syndrome at a single institution. J Pediatr Hematol Oncol. 2009;31: 81-4.

31. Ding Y, Ge Y. Anti-melanoma differentiation-associated gene 5 antibody-positive dermatomyositis complicated with macrophage activation syndrome. Ther Adv Chronic Dis. 2022;13: 20406223221098128.

32. Kawasumi H, Katsumata Y, Nishino A, Hirahara S, Kawaguchi Y, Kuwana M, et al. Association of Serum Soluble CD163 with Polymyositis and Dermatomyositis, Especially in Anti-MDA5 Antibody-positive Cases. J Rheumatol. 2018;45(7):947–55.

33. Coca A, Bundy KW, Marston B, Huggins J, Looney RJ. Macrophage activation syndrome: serological markers and treatment with anti-thymocyte globulin. Clin Immunol 2009;132(1):10–8.

34. Peng QL, Zhang YL, Shu XM, Yang HB, Zhang L, Chen F, et al. Elevated Serum Levels of Soluble CD163 in Polymyositis and Dermatomyositis: Associated with Macrophage Infiltration in Muscle Tissue. J Rheumatol. 2015; 42(6):979-87.

35. Gono T, Sato S, Kawaguchi Y, Kuwana M, Hanaoka M, Katsumata Y, et al. Anti-MDA5 antibody, ferritin and IL-18 are useful for the evaluation of response to treatment in interstitial lung disease with anti-MDA5 antibody-positive dermatomyositis. Rheumatology (Oxford). 2012;51(9):1563-70.

36. Nagashima T, Kamata Y, Iwamoto M, Okazaki H, Fukushima N, Minota S. Liver dysfunction in anti-melanoma differentiation-associated gene 5 antibody-positive patients with dermatomyositis. Rheumatol Int. 2019;39(5): 901-909.

37. Ibarra MF, Klein-Gitelman M, Morgan E, Proytcheva M, Sullivan C, Morgan G, et al. Serum neopterin levels as a diagnostic marker of hemophagocytic lymphohistiocytosis syndrome. Clin Vaccine Immunol. 2011;18(4):609-14.

38. Khojah A, Morgan G, Pachman LM. Clues to Disease Activity in Juvenile Dermatomyositis: Neopterin and Other Biomarkers. Diagnostics (Basel). 2021;12(1):8.

39. Li S, Li W, Jiang W, He L, Peng Q, Wang G, et al. The Efficacy of Tocilizumab in the Treatment of Patients with Refractory Immune-Mediated Necrotizing Myopathies: An Open-Label Pilot Study. Front Pharmacol. 2021;12: 635654

40. Kishida D, Sakaguchi N, Ueno KI, Ushiyama S, Ichikawa T, Yoshinaga T, et al. Macrophage activation syndrome in adult dermatomyositis: a case-based review. Rheumatol Int. 2020;40(7):1151-1162.

41. Eloseily EM, Weiser P, Crayne CB, Haines H, Mannion ML, Stoll ML, et al. Benefit of Anakinra in Treating Pediatric Secondary Hemophagocytic Lymphohistiocytosis. Arthritis Rheumatol. 2020;72(2):326-334.
